# Microbial Food Safety and Antimicrobial Resistance in Foods: A Dual Threat to Public Health

**DOI:** 10.3390/microorganisms13071592

**Published:** 2025-07-06

**Authors:** Ayman Elbehiry, Eman Marzouk, Adil Abalkhail, Husam M. Edrees, Abousree T. Ellethy, Abdulaziz M. Almuzaini, Mai Ibrahem, Abdulrahman Almujaidel, Feras Alzaben, Abdullah Alqrni, Akram Abu-Okail

**Affiliations:** 1 Department of Public Health, College of Applied Medical Sciences, Qassim University, P.O. Box 6666, Buraydah 51452, Saudi Arabia; e.marzouk@qu.edu.sa (E.M.);; 2 Department of Physiology, Faculty of Medicine, University of Tabuk, Tabuk 71491, Saudi Arabia; 3 Department of Basic Oral Sciences and Dental Education, College of Dentistry, Qassim University, Buraydah 51452, Saudi Arabia; 4 Department of Veterinary Preventive Medicine, College of Veterinary Medicine, Qassim University, Buraydah 51452, Saudi Arabia; 5 Department of Public Health, College of Applied Medical Science, King Khalid University, Abha 61421, Saudi Arabia; 6 Department of Food Service, King Fahad Armed Hospital, Jeddah 23311, Saudi Arabia; 7 Department of Family Medicine, King Fahad Armed Hospital, Jeddah 23311, Saudi Arabia; 8 Department of Pathology and Laboratory Diagnosis, College of Veterinary Medicine, Qassim University, Buraydah 51452, Saudi Arabia

**Keywords:** antimicrobial resistance (AMR), foodborne pathogens, One Health, surveillance systems, microbial food safety, Public Health

## Abstract

The intersection of microbial food safety and antimicrobial resistance (AMR) represents a mounting global threat with profound implications for public health, food safety, and sustainable development. This review explores the complex pathways through which foodborne pathogens—such as *Salmonella* spp., *Escherichia coli* (*E. coli*), *Listeria monocytogenes* (*L. monocytogenes*), and *Campylobacter* spp.—acquire and disseminate resistance within human, animal, and environmental ecosystems. Emphasizing a One Health framework, we examine the drivers of AMR across sectors, including the misuse of antibiotics in agriculture, aquaculture, and clinical settings, and assess the role of environmental reservoirs in sustaining and amplifying resistance genes. We further discuss the evolution of surveillance systems, regulatory policies, and antimicrobial stewardship programs (ASPs) designed to mitigate resistance across the food chain. Innovations in next-generation sequencing, metagenomics, and targeted therapeutics such as bacteriophage therapy, antimicrobial peptides (AMPs), and CRISPR-based interventions offer promising alternatives to conventional antibiotics. However, the translation of these advances into practice remains uneven, particularly in low- and middle-income countries (LMICs) facing significant barriers to diagnostic access, laboratory capacity, and equitable treatment availability. Our analysis underscores the urgent need for integrated, cross-sectoral action—anchored in science, policy, and education—to curb the global spread of AMR. Strengthening surveillance, investing in research, promoting responsible antimicrobial use, and fostering global collaboration are essential to preserving the efficacy of existing treatments and ensuring the microbiological safety of food systems worldwide.

## 1. Introduction

**Figure 1:**
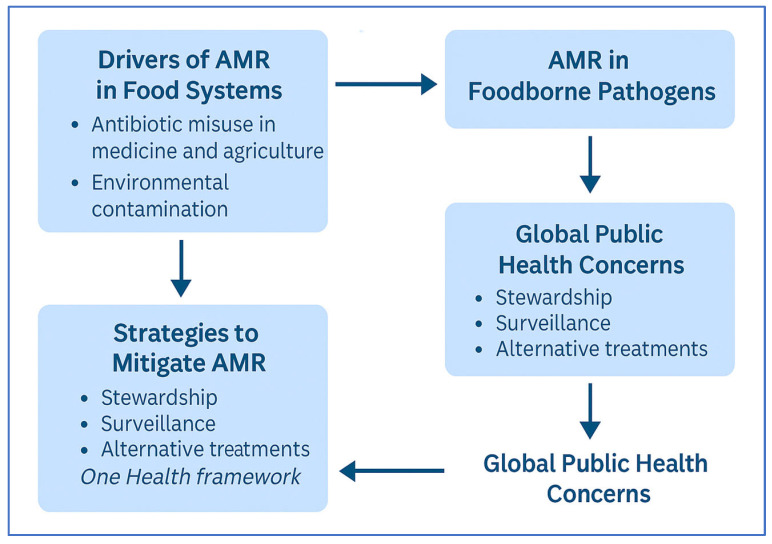
Conceptual diagram illustrating the progression from drivers of AMR in food systems—including antibiotic misuse and environmental contamination—to the emergence of AMR in foodborne pathogens, associated global public health concerns, and key mitigation strategies within a One Health framework.

Ensuring the microbial safety of food remains a paramount concern in public health, as foodborne illnesses continue to pose significant challenges globally. Pathogens such as *Salmonella* spp., *Escherichia coli* (*E. coli*), *Listeria monocytogenes* (*L. monocytogenes*), and *Campylobacter* spp. are frequently implicated in outbreaks, which are often linked to contaminated meat, dairy, and ready-to-eat (RTE) products [1,2]. The emergence and proliferation of antimicrobial-resistant (AMR) strains among these pathogens exacerbate the issue, rendering conventional treatments less effective and leading to persistent infections [2,3]. As illustrated in Figure 1, key drivers such as antimicrobial misuse and environmental contamination contribute to the development of AMR in foodborne pathogens. This leads to significant global public health concerns and necessitates mitigation strategies within a One Health framework. The figure provides a conceptual summary of these pathways.

The misuse and overuse of antibiotics in both human medicine and agriculture have accelerated the development of AMR. In the agricultural sector, antibiotics are frequently employed not only for therapeutic purposes but also for growth promotion and disease prevention in livestock [4,5]. This practice contributes to the emergence of multidrug-resistant (MDR) bacteria, which can be transmitted to humans through the food chain [6,7,8]. Notably, MDR strains of *Staphylococcus aureus* (*S. aureus*), *E. coli*, and *Salmonella* have been isolated from meat and dairy products, posing significant health risks [9,10].

RTE foods are particularly concerning, as they often bypass cooking processes that would eliminate harmful bacteria. Studies have detected MDR pathogens in various RTE products, highlighting the need for stringent hygiene practices and monitoring [11,12]. The World Health Organization (WHO) recognizes AMR as one of the top global public health threats, estimating that by 2050, AMR could cause up to 10 million deaths annually if no action is taken [13,14]. This underscores the urgency of addressing AMR through a One Health approach that considers the interconnectedness of human, animal, and environmental health [15,16]. Lammie and Hughes [17] identified AMR as a complex global health challenge closely linked to food systems and emphasized that addressing it requires a multifaceted strategy and a One Health approach involving improved communication, cooperation, and collaboration across sectors.

In recent years, the intensification of global food production and international trade has heightened the complexity of microbial risk management across the food supply chain. The increasing movement of agricultural goods across borders facilitates the transboundary spread of antimicrobial-resistant bacteria and resistance genes [9,10]. Environmental factors, including the contamination of water sources used in irrigation or livestock production, contribute to the persistence and amplification of AMR in food ecosystems [11]. Moreover, metagenomic studies have revealed that resistance determinants are not only prevalent in clinically relevant bacteria but also in commensal organisms, which may act as reservoirs and vectors for horizontal gene transfer [15,18]. This underscores the need for comprehensive monitoring strategies that go beyond routine pathogen surveillance to include resistome and mobilome analyses across the entire farm-to-fork continuum.

Technological advances offer promising avenues for addressing the intertwined challenges of foodborne pathogens and AMR. Novel molecular approaches, including real-time PCR, next-generation sequencing (NGS), and metagenomic shotgun sequencing, have significantly improved the speed and sensitivity of pathogen detection and resistance profiling in complex food matrices [2,19]. These tools not only facilitate the early detection of contamination but also enable the identification of emerging resistance traits before they become widespread. Furthermore, innovative control measures such as bacteriophage therapy, antimicrobial peptides (AMPs), and microbiome modulation are being explored as alternatives to conventional antibiotics [8,20]. However, the implementation of these approaches requires coordinated efforts among policymakers, food producers, and public health authorities to ensure feasibility, safety, and consumer acceptance.

Efforts to combat AMR in the food sector include implementing antimicrobial stewardship programs, enhancing the surveillance of resistant strains, and promoting research into alternative treatments [21,22]. Additionally, public education on proper food handling and the risks associated with antibiotic misuse is crucial [20,23]. Recent studies have also emphasized the role of consumers in mitigating AMR by making informed choices and advocating for responsible antibiotic use in food production [24,25,26]. This review aims to explore the current state of microbial safety and AMR in foods, examining the prevalence of MDR pathogens in meat, dairy, and RTE products, and discussing strategies to mitigate these risks.

Moreover, this review addresses a critical gap in the current literature by integrating the implications of AMR for food safety. While previous reviews have focused primarily on AMR’s impact on human and animal health, this work uniquely highlights how AMR in foodborne pathogens threatens food availability, access, and stability. It synthesizes recent data on resistant strains in foods, contamination sources, prevention strategies, detection methodologies, and legislation, all within the context of food safety. The manuscript presents a cohesive, evidence-based narrative linking data, analysis, and practical strategies under a unified One Health framework, concluding with key implications for global food safety.

## 2. Methodology

This narrative review was conducted to synthesize current knowledge on foodborne AMR under the One Health framework. Literature searches were performed in PubMed, Scopus, and Web of Science databases up to 2025. The following keywords were used in various combinations: AMR, food safety, foodborne pathogens, One Health, surveillance, AMR policy, and control strategies. Inclusion criteria comprised peer-reviewed articles, systematic reviews, meta-analyses, surveillance reports, and major international agency documents published in English. Priority was given to studies from the last 10 years, but older seminal works were also considered where relevant. Data extraction focused on AMR prevalence, drivers, surveillance, mitigation strategies, and policy frameworks. The selection process was performed by the authors independently, with consensus achieved through discussion.

## 3. Foodborne Pathogens and the Burden of AMR

**Figure 2:**
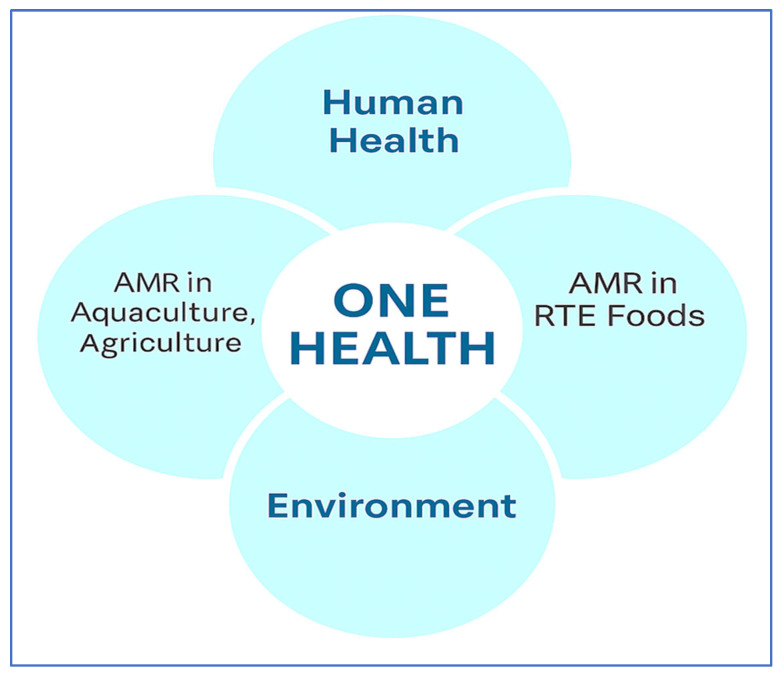
One Health schematic illustrating the pathways of AMR transmission between animals, food, environment, and humans. Key resistance mechanisms, such as plasmid-mediated transfer and environmental reservoirs, are adapted from Hendriksen et al. [32] and Van Boeckel et al. [30]. The figure highlights the interconnectedness of foodborne AMR across sectors.

Foodborne pathogens such as *Salmonella* spp., *E. coli*, *L. monocytogenes*, and *Campylobacter* spp. are major contributors to foodborne illnesses worldwide. According to the Antimicrobial Resistance Collaborators [27], bacterial AMR was directly responsible for 1.27 million deaths and contributed to nearly 5 million global deaths in 2019. These pathogens, particularly when resistant to multiple antibiotics, pose serious health risks due to prolonged illness, increased hospitalization, and higher mortality [28,29]. The misuse and overuse of antibiotics in agriculture play a substantial role in accelerating resistance. Farmers often use antimicrobials not only therapeutically but also for growth promotion and prophylaxis, especially in poultry and livestock [5,30]. These practices create reservoirs of MDR bacteria in the food chain, including *E. coli* and *Salmonella*, which have been isolated from meat, dairy, and RTE products [6,31]. Figure 2 presents a schematic of the One Health framework, illustrating the interconnected roles of human health, animal health (including aquaculture and agriculture), food systems, and the environment in addressing AMR. The figure highlights how these sectors contribute to and are impacted by AMR challenges in food production and safety.

Aquaculture is a rapidly growing sector in global food production and is increasingly being recognized as a hotspot for AMR. The intensive use of antibiotics in fish and shrimp farming to prevent disease and enhance productivity fosters the selection of resistant bacterial strains. Deng et al. [33] analyzed 23,165 antimicrobial resistance gene (ARG) contigs from aquaculture isolates in 75 countries. They reported that *blaCMY-2* occurred in approximately 10–15% of samples, cphA in 30–35%, and mcr-3 in 5–10%, highlighting the widespread presence of mobile ARGs in aquaculture environments, which poses substantial risks for transfer to human pathogens. Yuan et al. [34] corroborated this by documenting widespread contamination with antibiotics, resistant bacteria, and ARGs across water, sediment, and aquatic species. Similarly, Hossain et al. [35] identified elevated antibiotic concentrations in Bangladeshi aquaculture environments, posing ecological and public health risks. Together, these findings underscore the urgent need for surveillance, judicious antimicrobial use, and effective regulatory frameworks to contain AMR emergence from aquaculture systems.

*L. monocytogenes*, a significant concern in cold-stored RTE foods, has exhibited resistance to clinically important antimicrobials including ciprofloxacin and erythromycin, though resistance to first-line treatments like penicillin G and tetracycline remains relatively rare [36]. Similarly, *Campylobacter jejuni* has demonstrated widespread fluoroquinolone resistance, particularly in poultry systems, driven by chromosomal mutations and horizontal gene transfer [37,38]. Surveillance reports by the European Food Safety Authority (EFSA) [39] and the U.S. Centers for Disease Control and Prevention [40] confirm a significant increase in AMR, as demonstrated by rising multidrug-resistant *Salmonella* and extended-spectrum β-lactamase (ESBL)-producing *E. coli* rates in food products globally [41]. Notably, MDR *Salmonella enterica* (*S. enterica*) and ESBL-producing *E. coli* have been found in poultry, pork, and beef, with frequent resistance to ciprofloxacin and third-generation cephalosporins [42,43]. In support of these findings, Shafiq et al. [44] documented the presence of ESBL-encoding plasmids along China’s pork production chain, illustrating the global scale of this threat.

Environmental pathways also contribute significantly to AMR transmission. Zhang et al. [45] demonstrated that ARGs from manure-amended soils can transfer to the microbial communities of vegetables such as lettuce, revealing a direct route for resistance to enter the food chain. In addition, studies by Zhu et al. [46] and Berendonk et al. [11] emphasized the role of agricultural runoff and wastewater as reservoirs and vectors of ARGs in both environmental and agricultural ecosystems. Resistance genes such as *blaCTX-M*, *mcr-1*, and *tetA* are not only found in pathogens but are also common among commensal bacteria, often carried on mobile genetic elements that promote interspecies dissemination [16]. Recently, Keet and Rip [47] reported MDR *L. monocytogenes* strains isolated from raw meats and ready-to-eat foods in the Western Cape, South Africa, underscoring the regional challenges in managing antimicrobial resistance in foodborne pathogens.

An increasing burden of AMR in foodborne pathogens: EFSA and ECDC [41] estimate approximately 7% of confirmed human salmonellosis and 17% of campylobacteriosis cases in the EU are caused by resistant strains. In 2019, there were over 300 confirmed outbreaks linked to multidrug-resistant (MDR) Salmonella in EU member states, resulting in approximately 2500 reported hospitalizations. To curb the growing AMR burden in food systems, a comprehensive One Health approach is essential—one that integrates food safety protocols, veterinary antimicrobial stewardship, and environmental controls. Non-traditional interventions such as bacteriophage therapy [48] and bacteriocins [49] show promise as future alternatives to antibiotics. Public education, policy enforcement, and consumer-driven advocacy for responsible antibiotic use remain pivotal elements of long-term containment strategies [49].

## 4. One Health Approach to Mitigating AMR

**Figure 3:**
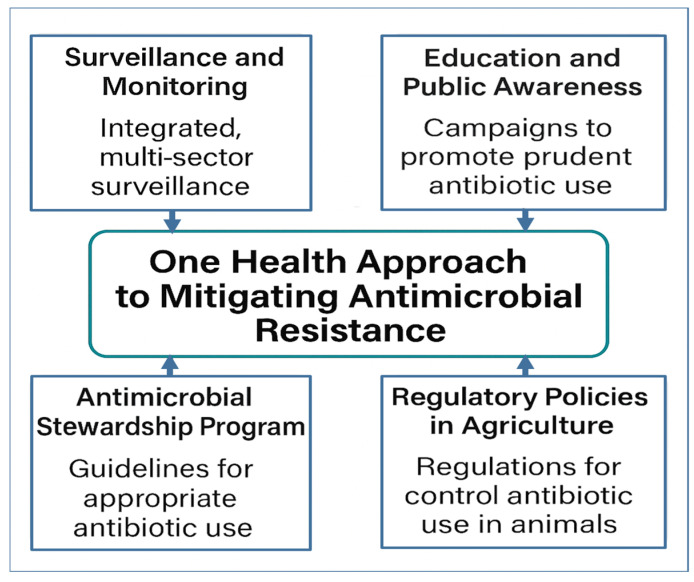
One Health approach to mitigating AMR. The diagram illustrates the integration of surveillance, education, stewardship, and regulatory policies required to control AMR across human, animal, and environmental sectors.

AMR is a multifaceted global health challenge that transcends the boundaries of human medicine, encompassing animal health, agriculture, and environmental ecosystems. The One Health approach, which recognizes the interconnectedness of these domains, has emerged as a pivotal strategy in addressing the complex dynamics of AMR. As illustrated in Figure 3, the One Health approach provides a comprehensive framework that integrates human, animal, and environmental health sectors to address the multifaceted nature of AMR. This model guides the subsequent sections by mapping key drivers, surveillance mechanisms, interventions, innovations, educational strategies, and global policy responses across all domains.

### 4.1. Conceptual Framework of One Health in AMR

The One Health paradigm emphasizes the integration of human, animal, and environmental health sectors to develop comprehensive strategies against AMR. This approach acknowledges that the misuse and overuse of antimicrobials in any of these sectors can contribute to the emergence and dissemination of resistant pathogens, necessitating coordinated interventions across all domains.

### 4.2. Drivers of AMR Across Sectors

#### 4.2.1. Human Health Sector

In the human health domain, inappropriate antibiotic prescribing remains a major contributor to the emergence of AMR. Numerous studies have revealed that a substantial proportion of antibiotic prescriptions are unnecessary, often issued for self-limiting viral infections or under patient pressure. In a systematic review, Sijbom et al. [50] identified multiple determinants influencing inappropriate antibiotic prescriptions in primary care, including diagnostic uncertainty, perceived patient expectations, and lack of rapid diagnostic tools. Browne et al. [51] further demonstrated that global antibiotic consumption rose sharply between 2000 and 2018, particularly in LMICs where access and stewardship programs are often limited. In a pivotal U.S. study, Fleming-Dutra et al. [52] reported that approximately 30% of outpatient antibiotic prescriptions were unnecessary, underscoring the systemic overuse in ambulatory care.

To address the persistent problem of antibiotic misuse, ASPs have been widely implemented in clinical settings. In a national survey of U.S. hospitals, Pollack et al. [53] reported that only 39% had fully adopted all seven Centers for Disease Control and Prevention (CDC) Core Elements of Hospital ASPs as of 2014, underscoring both progress and the need for ongoing institutional commitment. These core elements—ranging from leadership commitment and accountability to education and tracking—have since become the standard framework for promoting judicious antibiotic use in healthcare facilities. Complementary to this, the CDC continues to update and disseminate stewardship guidelines through its Core Elements of Hospital Antibiotic Stewardship Programs [54].

Beyond the frequency of antibiotic use, inappropriate selection of antibiotics for common infections is also a growing concern. Ardillon et al. [55] conducted a multicentric cohort study in three LMICs and found high rates of inappropriate antibiotic prescribing in outpatient pediatric care. The study highlighted that such practices not only contribute to resistance but also increase the risk of treatment failure and adverse outcomes, particularly in settings with limited diagnostic capacity. Their findings reinforce the need for tailored stewardship interventions, especially in community-level primary healthcare systems.

#### 4.2.2. Animal Health and Agriculture

The agricultural sector, particularly intensive livestock farming, has been identified as a major contributor to AMR due to the prophylactic and growth-promoting use of antibiotics. Van Boeckel et al. [56] reported that approximately 73% of all antimicrobials sold globally are used in animals raised for food. This extensive use facilitates the selection of resistant bacteria, which can be transmitted to humans through direct contact or the consumption of animal products. Recent projections indicate that, under a business-as-usual scenario, global antibiotic use in livestock could reach approximately 143,481 tons by 2040, representing a 29.5% increase from the 2019 baseline of around 110,777 tons. This underscores the urgency for coordinated efforts to reduce antibiotic use intensity and manage livestock biomass effectively [57].

In low- and middle-income countries, the situation is particularly concerning. A study analyzing 901 point-prevalence surveys revealed that China and India are significant hotspots for AMR in animals, with emerging hotspots in Brazil and Kenya. From 2000 to 2018, the proportion of antimicrobials showing resistance above 50% increased from 15% to 41% in chickens and from 13% to 34% in pigs [56]. The misuse of antibiotics in animal agriculture not only affects animal health but also poses a significant threat to human health. The CDC highlights that antimicrobial-resistant germs can spread between animals and humans through various pathways, including direct contact, consumption of contaminated food, and environmental routes such as water runoff from farms [54].

Furthermore, the environmental impact of antibiotic use in agriculture cannot be overlooked. Antibiotics and antifungals used in animals can contaminate the environment through animal waste, which may carry drug residues and resistant germs. This contamination can affect soil and water sources, contributing to the development and spread of resistant germs in the environment [54]. To mitigate these risks, it is essential to implement stringent regulations on antibiotic use in agriculture, promote good animal husbandry practices, and enhance surveillance of antibiotic use and resistance patterns in the agricultural sector. Adopting a One Health approach that integrates human, animal, and environmental health strategies is crucial in combating the spread of AMR.

#### 4.2.3. Environmental Sector

The environment acts as both a reservoir and a conduit for AMR, facilitating the dissemination of resistant bacteria and genes through soil, water, and air. Pharmaceutical effluents, agricultural runoff, and improper waste disposal introduce antibiotics and resistant organisms into environmental compartments, accelerating the spread of resistance. Berendonk et al. [11] highlighted the persistence of ARGs in aquatic and terrestrial ecosystems, advocating for environmental surveillance as a critical component of AMR mitigation strategies. Recent global assessments have reinforced the environmental dimension of AMR. The United Nations Environment Programme has stressed that pollution from human activities—including pharmaceutical manufacturing, agriculture, and waste mismanagement—is a key driver of AMR emergence and transmission. This underscores the necessity for pollution control and ecosystem health in AMR policy [58].

Aquatic systems, in particular, have emerged as hotspots for ARG accumulation. Cheung et al. [59] conducted a scoping review and found that fecal pollution in surface waters leads to significant concentrations of resistant bacteria, posing ecological and public health risks. The review emphasized the importance of adequate sanitation infrastructure and improved wastewater treatment technologies in controlling the spread of resistance. Furthermore, climate change has been increasingly associated with AMR dynamics. Fernández Salgueiro et al. [60] reviewed the influence of environmental factors such as rising temperatures, flooding, and droughts on microbial evolution and horizontal gene transfer. These climate-related stressors are believed to exacerbate AMR propagation, especially in vulnerable ecosystems.

Furthermore, the presence of antibiotic residues in the environment has been recognized as a driver for AMR. Larsson and Flach [61] discussed the impact of antibiotic pollution on the emergence of resistant bacteria, emphasizing the need for stringent regulations on antibiotic disposal and wastewater treatment. Implementing such measures is crucial to prevent the proliferation of resistance genes in environmental settings. Together, these findings highlight the need to include the environmental sector in AMR surveillance and response strategies. A One Health approach that integrates environmental protection, sanitation infrastructure, and antibiotic waste regulation is essential to prevent the unchecked spread of resistance beyond clinical and agricultural settings.

### 4.3. Surveillance and Monitoring

Surveillance and monitoring are critical pillars in addressing AMR, enabling early detection of resistance trends, identifying emerging threats, and evaluating the effectiveness of interventions. A robust surveillance system must adopt a One Health approach, integrating data from human health, animal health, agriculture, and the environment to comprehensively map resistance dynamics and inform policy decisions. The Global Antimicrobial Resistance Surveillance System (GLASS), launched by the WHO, exemplifies this integrated framework. GLASS promotes standardized collection and reporting of AMR and antimicrobial consumption data across member states, thereby enhancing the comparability and utility of global surveillance outputs [62]. As of the latest GLASS report, over 120 countries are enrolled, contributing data on priority pathogens such as *E. coli*, *Klebsiella pneumoniae*, *Salmonella* spp., and *S. aureus*, alongside their resistance patterns and treatment challenges.

In Europe, the European AMR Surveillance Network, managed by the European CDC, focuses on AMR surveillance in invasive bacterial infections. The network utilizes data from over 30 countries to generate harmonized resistance trends, offering policymakers timely insights into the effectiveness of national and EU-wide antimicrobial stewardship programs [63]. Complementing this, the European Surveillance of Veterinary Antimicrobial Consumption collects data on veterinary antimicrobial sales, enabling assessment of trends in antimicrobial usage in food-producing animals [64]. In the United States, the National AMR Monitoring System provides integrated surveillance across human, animal, and food sectors. Jointly coordinated by the CDC, FDA, and United States Department of Agriculture (USDA), National Antimicrobial Resistance Monitoring System (NARMS) tracks resistance in pathogens such as *Salmonella*, *Campylobacter*, and *E. coli* from clinical, retail meat, and animal sources. These findings guide regulatory action, including the restriction of critical antimicrobials in livestock [65].

For LMICs, surveillance implementation often faces logistical and technical barriers. To bridge these gaps, the Fleming Fund, funded by the UK government, supports laboratory strengthening, data harmonization, and human–animal surveillance integration in over 20 LMICs [66]. Similarly, the Food and Agriculture Organization of the United Nations (FAO)’s ATLASS tool (Assessment Tool for Laboratories and AMR Surveillance Systems) provides a structured approach to evaluating and enhancing national AMR surveillance in food and agriculture systems [67]. Environmental surveillance is an emerging frontier in AMR monitoring. Wastewater-based epidemiology has shown promise as a cost-effective method to capture community-wide AMR burdens. A landmark study by Hendriksen et al. [32] demonstrated that metagenomic analysis of urban sewage could accurately reflect local resistance gene prevalence and trends, offering a scalable model for global surveillance.

Advances in whole-genome sequencing (WGS) and bioinformatics have further revolutionized AMR tracking. By enabling high-resolution pathogen typing and identification of resistance genes, WGS supports outbreak detection, resistance mechanism elucidation, and real-time tracking of resistant clones. Several national AMR programs, including those in the UK, Australia, and the Philippines, have incorporated WGS into their routine surveillance, enhancing data precision and intervention targeting [68]. Nonetheless, critical challenges persist: data fragmentation, limited real-time sharing, disparities in capacity, and a lack of standardized methodologies across sectors and regions. To address these gaps, international collaboration, open-access data platforms, harmonized protocols, and investment in surveillance infrastructure remain essential.

### 4.4. Interventions and Strategies

#### 4.4.1. Antimicrobial Stewardship Programs (ASPs)

ASPs are structured, evidence-based initiatives aimed at promoting the responsible use of antimicrobials to achieve optimal clinical outcomes while minimizing the selection and spread of antimicrobial-resistant organisms. These programs are essential pillars of national and global strategies to combat AMR, with direct implications for both public health and food system resilience. The primary goals of ASPs include ensuring the appropriate selection, dosage, route, and duration of antimicrobial therapy, thereby reducing unnecessary or inappropriate use. These programs often operate through multidisciplinary teams—comprising infectious disease specialists, clinical microbiologists, pharmacists, and infection control experts—who develop local antibiotic guidelines, monitor prescribing behavior, and provide prescriber feedback.

Dyar et al. [69] showed that ASP implementation across European hospitals led to a significant reduction in broad-spectrum antibiotic use and improved compliance with prescribing standards. A systematic review by Baur et al. [70] covering 32 hospital-based ASPs revealed a 35% reduction in antibiotic consumption and a 10% decline in AMR rates, with no adverse effects on patient mortality or clinical outcomes. In the United States, ASPs are now mandated in all acute care hospitals under federal regulations. The CDC’s Core Elements of Hospital Antibiotic Stewardship Programs guide healthcare institutions in building sustainable ASP infrastructure, emphasizing leadership support, tracking, reporting, and staff education [54]. Importantly, the relevance of ASPs extends far beyond hospital walls. In veterinary medicine and food animal production, ASPs aim to curtail the overuse and misuse of antimicrobials that contribute to the emergence of resistant foodborne pathogens. The use of critically important antimicrobials in livestock—often for growth promotion or prophylaxis—has been strongly linked to AMR-positive isolates of *Salmonella*, *E. coli*, *L. monocytogenes*, and *Campylobacter* detected in meat and dairy products. Accordingly, stewardship practices in the agricultural sector are increasingly viewed as central to the prevention of foodborne AMR transmission.

In LMICs, ASP implementation faces logistical and economic barriers. Nevertheless, promising examples have emerged. In India, several studies have documented the progress and impact of ASPs. Singh et al. [71] demonstrated that ASP implementation in a tertiary care hospital significantly reduced the use of high-end antibiotics and improved patient outcomes. Debnath et al. [72] reported a successful introduction of ASPs in primary and secondary care hospitals, emphasizing that even resource-limited settings can benefit from tailored interventions. Sahni et al. [73] conducted a systematic review highlighting the gaps in ASP implementation across Indian healthcare facilities and calling for strengthened institutional policies, local antibiogram development, and routine training of healthcare personnel. When coupled with infection prevention and control measures, ASPs have been shown to amplify reductions in resistant infections. Furthermore, digital tools—such as real-time decision support systems, electronic prescribing, and automated alerts—are increasingly enhancing the efficacy and scalability of stewardship initiatives. In conclusion, ASPs represent a foundational strategy in the One Health response to AMR. Expanding their reach beyond clinical settings to include animal health and food production is essential to controlling the spread of resistant bacteria, protecting food safety, and preserving antibiotic efficacy for future generations.

#### 4.4.2. Regulatory Policies in Agriculture

The regulation of antimicrobial use in agriculture is a critical intervention in the global effort to combat AMR, especially given the established link between antibiotic use in food-producing animals and the emergence of resistant bacteria in the human population. These regulations aim to restrict non-therapeutic uses of antibiotics, improve veterinary oversight, and promote responsible antimicrobial practices across the food production chain. A landmark intervention was the European Union (EU)’s 2006 ban on the use of antibiotics as growth promoters in livestock. This policy was followed by substantial reductions in resistance rates among zoonotic bacteria such as *Salmonella* and *Campylobacter*, with no negative impact on animal productivity when good farming practices were maintained [20]. Countries like Denmark and the Netherlands have further advanced these efforts by enforcing strict antimicrobial usage monitoring systems, mandating veterinary prescriptions, and incentivizing low-antibiotic farming [30,74].

In the United States, the FDA issued Guidance for Industry #213, which led to the withdrawal of medically important antimicrobials for growth promotion and introduced veterinary oversight for therapeutic use. The FDA’s 2022 report shows a continued decline in overall sales of such antibiotics in food animals, reflecting the positive impact of regulatory reforms [75]. In emerging economies, the enforcement of antimicrobial policies remains inconsistent. However, countries like China and India have begun to implement restrictions on the use of critically important antimicrobials. For example, China banned colistin as a feed additive following the discovery of the plasmid-mediated colistin resistance gene *mcr-1*, which raised global concern due to its potential for rapid spread [76]. India has also issued national action plans and advisories to regulate antibiotic use in poultry and aquaculture, although implementation and surveillance remain in early stages [77].

International agencies, including the WHO, the FAO, and the World Organization for Animal Health (WOAH), advocate for harmonized global actions. These include phasing out antibiotics for growth promotion, banning over-the-counter antimicrobial sales, and establishing surveillance systems for antimicrobial use and resistance in the animal sector. Ultimately, effective regulatory policies—when combined with enhanced farm biosecurity, vaccination programs, and improved animal husbandry—can substantially reduce antibiotic dependence in food production, lower AMR transmission risk to humans, and contribute to both public health protection and food system sustainability.

#### 4.4.3. Environmental Management

The environment is a critical but often overlooked component in the transmission and persistence of AMR. Environmental compartments—such as soil, surface water, groundwater, and sediments—act as reservoirs and conduits for antimicrobial residues, resistant bacteria, and resistance genes originating from human, animal, and industrial sources. To mitigate environmental contamination, a multipronged approach is essential. One key strategy is enhancing wastewater treatment infrastructure, particularly in hospitals, pharmaceutical manufacturing sites, and agricultural operations. Conventional wastewater treatment plants are not specifically designed to remove antibiotics or resistance genes, allowing these elements to enter natural water bodies and spread across ecosystems [78]. Upgrading treatment processes with advanced oxidation technologies, membrane filtration, and constructed wetlands can significantly reduce the environmental AMR burden.

Another important intervention involves regulating pharmaceutical discharges. Industrial effluents from drug manufacturing facilities—especially in countries with weak environmental oversight—are often heavily contaminated with antibiotics, creating hotspots for resistance gene selection and horizontal gene transfer [79]. Implementing strict discharge limits, enforcing zero-liquid-discharge technologies, and ensuring routine environmental monitoring can curtail these emissions. Sustainable agricultural practices also play a crucial role in minimizing the release of antibiotics and resistant microbes into the environment. This includes the safe management of animal manure, composting, limiting antibiotic use in aquaculture, and promoting integrated pest and disease management. Improved handling of farm waste reduces the leaching of contaminants into soils and water bodies, thereby lowering the environmental load of AMR determinants.

Larsson et al. [79] emphasized the necessity of global environmental regulations as part of a One Health response to AMR. They argued for the inclusion of AMR-relevant targets in environmental protection frameworks and greater collaboration between the health, environment, and agriculture sectors. Recent initiatives, such as the AMR Industry Alliance’s manufacturing framework and the Stockholm Statement on Pharmaceuticals in the Environment, advocate for sustainable antibiotic production and improved environmental accountability. Ultimately, reducing environmental contributions to AMR requires coordinated global action—combining policy enforcement, technological innovation, industry responsibility, and international funding to strengthen environmental stewardship as a central pillar in the AMR response.

### 4.5. Research and Innovation

Continued investment in research and innovation is essential for tackling the growing challenge of AMR and safeguarding microbial food safety. Traditional antibiotics are losing their efficacy at an alarming rate, and the development pipeline for new antimicrobials remains limited. In response, research efforts have increasingly focused on novel therapeutics, next-generation diagnostics, and genomic tools that support precision intervention. Bacteriophage (phage) therapy has re-emerged as a promising alternative to conventional antibiotics, particularly against MDR pathogens. Phages are viruses that specifically target and lyse bacterial cells without affecting the host microbiota or contributing to resistance selection. Recent studies have demonstrated the successful use of phage cocktails to treat severe MDR infections in humans, and their potential application in veterinary medicine and food safety is gaining interest [80]. Phages can be applied to decontaminate food surfaces, control Listeria in dairy products, and reduce *Salmonella* in poultry processing.

AMPs, derived from microbial or host immune systems, represent another avenue of therapeutic innovation. These molecules exhibit broad-spectrum activity against bacteria, fungi, and viruses, and some are being explored for use in food preservation and livestock treatment with reduced resistance potential. Probiotics, prebiotics, and postbiotics are also under investigation for their role in enhancing gut health, competitively excluding pathogens, and reducing reliance on antibiotics in animal husbandry. For instance, certain strains of *Lactobacillus* and *Bacillus* have shown efficacy in improving animal productivity while minimizing pathogen shedding in feces. From a diagnostic standpoint, advances in genomics, metagenomics, and bioinformatics have significantly enhanced our ability to detect, characterize, and monitor resistance determinants. High-throughput sequencing platforms and resistome analyses allow researchers to track the evolution of resistance genes across human, animal, and environmental reservoirs. This is particularly important for understanding the transmission of AMR along the food chain and designing targeted interventions [81].

CRISPR-Cas systems offer highly specific antimicrobial activity by targeting resistance genes, providing a promising alternative to broad-spectrum antibiotics [82,83]. In diagnostics, biosensors, microfluidic devices, and AI-powered platforms enable real-time pathogen detection in food products, improving surveillance and response [84,85]. Advances in genomics and resistome analysis support tracking AMR transmission across the food chain and environment [81]. Global initiatives such as the Global Antibiotic Research and Development Partnership (GARDP) and Combating Antibiotic-Resistant Bacteria Biopharmaceutical Accelerator (CARB-X) support the development and equitable distribution of new antimicrobials and diagnostics, particularly in resource-limited settings. Sustained investment, cross-sector collaboration, and equitable access to innovation are essential to combat AMR and protect food systems.

While these innovative approaches offer promising solutions for AMR mitigation in food systems, their industrial implementation faces notable challenges. For bacteriophages, regulatory approval is evolving: Omar et al. [86] detail the current U.S. landscape, where only a few phage-based products (e.g., ListShield™, SalmoFresh) have received FDA/USDA approval. Challenges include establishing consistent manufacturing under Good Manufacturing Practices, ensuring formulation stability, and addressing industry concerns regarding integration into standard processing workflows. For AMPs, a recent review conducted by Pérez de la Lastra et al. [87] highlight their potential in food preservation through encapsulation and nanotechnology. However, large-scale production remains expensive and technically complex, with additional challenges related to maintaining activity in complex food matrices and securing regulatory acceptance. CRISPR-based tools likewise remain largely confined to research and pilot projects, with no current food industry applications. Acceptance by food producers and consumers hinges on a clear demonstration of efficacy, safety, and cost-competitiveness. To overcome these hurdles, harmonized regulatory frameworks, industrial-scale production technologies, and targeted stakeholder communication are essential [86,87].

### 4.6. Education and Public Awareness

Raising awareness about AMR across healthcare, agriculture, and the general public is a cornerstone of effective AMR mitigation. Misconceptions about antibiotic use and poor hygiene practices continue to fuel resistance, particularly in resource-limited settings. Targeted educational interventions among healthcare professionals improve prescribing behaviors and stewardship compliance. McCullough et al. [88] reported that structured awareness campaigns reduced misconceptions about resistance and promoted appropriate antibiotic use. Similarly, public health campaigns in high-income countries, such as those reviewed by Huttner et al. [89], led to measurable declines in outpatient antibiotic prescriptions.

In the livestock and poultry sectors, educational outreach and farmer training have shown strong impacts. A study by Chapot et al. [90] in Bangladesh demonstrated that farmer awareness programs reduced the misuse of antibiotics and improved animal husbandry practices. Global initiatives such as the World Antimicrobial Awareness Week, coordinated by WHO, have amplified public engagement through school-based programs, media outreach, and cross-sector collaboration [91]. Integrating AMR education into professional training, national curricula, and food safety regulations remains essential. Public awareness, driven by accurate communication and community participation, complements policy and research to ensure sustainable behavior change across sectors.

### 4.7. Global Collaboration and Policy Frameworks

AMR is a transboundary threat that requires coordinated action at local, national, and international levels. The interconnected nature of food systems, trade, human and animal health, and environmental exposures underscores the need for a unified, global response guided by strong policy frameworks and strategic collaboration. The WHO, in partnership with the FAO and the WOAH, has established the Tripartite Alliance, which serves as the cornerstone of the One Health Global Action Plan on AMR. This plan encourages nations to develop and implement National Action Plans tailored to local contexts while adhering to global objectives related to antimicrobial stewardship, surveillance, and capacity building [92]. To enhance food safety and reduce the spread of resistant pathogens through the food chain, these agencies promote regulations on antimicrobial use in agriculture, improvement of biosecurity and hygiene standards, and integrated AMR monitoring. The Codex Alimentarius Commission (Codex), co-managed by WHO and FAO, has issued Guidelines on Integrated Monitoring and Surveillance of Foodborne AMR [93], enabling member states to standardize practices in AMR data collection, risk assessment, and mitigation in food production.

Globally, funding initiatives such as the Fleming Fund, CARB-X, and the Global AMR R&D Hub are critical in strengthening AMR surveillance, diagnostics, and innovation, particularly in low- and middle-income countries. These programs support equitable access to laboratory infrastructure, early-stage research, and cross-sectoral data sharing to address gaps in implementation and capacity [66,94]. The GLASS, launched by WHO in 2015, now includes over 120 participating countries, standardizing AMR data in human health with plans to expand into food and environmental sectors [95]. Multilateral declarations by the United Nations, G7, and G20 have further positioned AMR as a global development and security threat, emphasizing the need for coordinated policy action. Effective frameworks must prioritize integration across health, agriculture, and the environment, while supporting countries to implement context-specific, enforceable, and sustainable AMR strategies.

Despite improvements in AMR surveillance globally, substantial gaps persist in foodborne AMR monitoring in vulnerable regions, including Latin America and sub-Saharan Africa. Severino et al. [96] conducted a systematic review of bacterial foodborne diseases in Central America and the Caribbean and identified critical deficiencies, including inadequate laboratory capacity, fragmented reporting systems, and limited integration of AMR data. In sub-Saharan Africa, Hendriksen et al. [32] highlighted that although genomic surveillance initiatives like SeqAfrica have enhanced AMR monitoring in human and animal sectors, One Health-aligned food AMR surveillance remains limited and fragmented. Addressing these gaps is essential for global AMR mitigation.

The integration of bioinformatics tools and predictive models into AMR surveillance is advancing rapidly. Metagenomic sequencing, combined with machine learning algorithms, allows for the detection and characterization of resistance genes directly from complex food and environmental samples without the need for culturing [32]. These approaches enable the identification of novel resistance determinants and provide early warnings for emerging threats. Machine learning models trained on genomic, epidemiological, and environmental data have been used to predict AMR outbreak risks and identify resistance hotspots in food systems [97]. Moreover, platforms such as the Global Sewage Surveillance Project and SeqAfrica have demonstrated the feasibility of using large-scale metagenomics for AMR monitoring across regions. Continued investment in bioinformatics capacity building, particularly in low- and middle-income countries, is critical to realize the full potential of these technologies for foodborne AMR surveillance. Table 1 provides a consolidated overview of key agencies and surveillance programs cited in this review, summarizing their primary roles in AMR mitigation and their interactions within One Health frameworks.

## 5. AMR and Food Safety: A Neglected Link in One Health

AMR in food systems represents an escalating challenge that threatens not only public health but also global food safety. The spread of resistant pathogens in food-producing environments can reduce access to safe, nutritious food, disrupt supply chains, and impose significant economic burdens on producers and consumers [30]. Food safety experts have emphasized that AMR in foodborne pathogens exacerbates food insecurity, particularly in LMICs, where food systems are most vulnerable [98].

### 5.1. Prevalence of Resistant Strains in Foods

A growing body of evidence demonstrates that foods of animal and aquatic origin serve as significant reservoirs for AMR bacteria, contributing to the global AMR burden and posing serious public health risks. In China, Tang et al. [99] examined retail pork and chicken meat samples from Zhejiang Province and found that 13% of *E. coli* isolates carried the colistin-resistance gene *mcr-1*; most of these isolates also produced extended-spectrum β-lactamases (ESBLs), indicating co-resistance to critical antibiotics. In Vietnam, Nhung et al. [100] reported that over 50% of *Salmonella* isolates from retail meat were MDR, with high levels of resistance to fluoroquinolones and extended-spectrum β-lactams, raising significant food safety concerns in the region. Phung et al. [101] also identified *L. monocytogenes* strains in RTE meat products, with several isolates displaying resistance to ampicillin and erythromycin—antibiotics commonly used in the clinical management of listeriosis.

In South Africa, Kayode and Okoh [102] analyzed *L. monocytogenes* isolates from RTE foods and reported high levels of AMR. Among 194 isolates, 53% were resistant to ceftriaxone, 61.9% to trimethoprim, and 62.9% to oxytetracycline. Notably, 83.5% of the isolates were multidrug-resistant, reflecting significant risks associated with RTE foods. In Egypt, Adel et al. [103] investigated *S. enterica* from retail meats and slaughterhouses, finding that 82.4% of isolates were multidrug-resistant. Among these, 41.2% produced ESBLs, and 67.6% carried plasmid-mediated quinolone resistance genes such as *qnrA*, *qnrB*, and *qnrS*, contributing to reduced efficacy of fluoroquinolone therapies. In Spain, Bort et al. [104] assessed *Campylobacter* spp. along the broiler production chain. Of 125 isolates, 97.6% exhibited resistance to at least one antibiotic, with particularly high levels of resistance to fluoroquinolones and macrolides—two key classes used in the treatment of campylobacteriosis. Collectively, these findings demonstrate that AMR hazards are widespread across the food chain and underscore the urgent need for integrated monitoring, prudent antimicrobial use in agriculture, and comprehensive food safety strategies at the global level.

### 5.2. Sources of Contamination

AMR contamination of foods can originate at multiple critical points across the agricultural, processing, and distribution continuum, posing significant challenges to food safety and public health. On farms, resistant bacteria can emerge and proliferate due to the widespread use of antibiotics not only for therapeutic purposes but also for prophylaxis and as growth promoters in food-producing animals [30]. Such practices exert selective pressure on microbial communities, facilitating the emergence and persistence of multidrug-resistant strains that can be transmitted through animal products. Crops may also serve as vehicles for AMR contamination. The application of animal manure as fertilizer, particularly when inadequately treated, introduces resistant bacteria and mobile genetic elements into agricultural soils [11]. Contaminated irrigation water—often carrying residues of antibiotics or resistant microorganisms from human and animal waste—represents an additional significant source of AMR dissemination in horticultural production [105]. These resistant bacteria can colonize plant surfaces or internal tissues, making their removal challenging during post-harvest handling.

At the slaughterhouse and food processing stages, poor hygiene practices can lead to direct cross-contamination of carcasses and meat products. Equipment, surfaces, and workers’ hands may act as vectors for the spread of resistant pathogens between contaminated and uncontaminated products [106]. The risk is compounded by the formation of biofilms on processing equipment, drains, and other hard-to-clean surfaces. Biofilms can harbor AMR bacteria and serve as reservoirs of resistance genes that persist despite standard sanitation measures [107]. These biofilms not only protect bacteria from environmental stressors but also enhance horizontal gene transfer among microbial populations. Furthermore, the distribution and retail environment can introduce additional contamination points. For example, inadequate temperature control, poor handling, and cross-contact between raw and ready-to-eat foods can facilitate the survival and spread of AMR bacteria [108]. Packaging materials and food contact surfaces may also contribute if not properly managed. Taken together, these diverse sources illustrate the complex and interconnected pathways through which AMR can enter and propagate within the food chain, underscoring the need for integrated surveillance and control measures at every stage from farm to fork.

### 5.3. Biochemical and Molecular Mechanisms of AMR in Foodborne Pathogens

AMR in foodborne pathogens is driven by several key biochemical and molecular mechanisms that enable bacteria to survive antibiotic exposure. Efflux pumps play a major role in multidrug resistance. The AcrAB-TolC system in *E. coli* and *Salmonella* and the CmeABC system in Campylobacter actively export antibiotics such as tetracyclines, macrolides, and fluoroquinolones, reducing their intracellular concentration [109]. Additional pumps such as EmrAB and MdfA contribute to resistance in *Salmonella* against a broader range of antibiotics.

β-lactamase production, particularly ESBLs like CTX-M and TEM variants, hydrolyze β-lactam antibiotics, rendering them ineffective. ESBL-producing *E. coli* and *Salmonella* have been frequently detected in retail meats and food handlers [110]. These enzymes are often plasmid-mediated and co-occur with other resistance genes. Plasmid-mediated quinolone resistance (PMQR) genes such as qnrA, qnrB, qnrS, and oqxAB protect DNA gyrase or enhance efflux, contributing to fluoroquinolone resistance in Enterobacterales from food sources [111].

Target site modifications also contribute to AMR. Mutations in the quinolone resistance-determining regions (QRDRs) of gyrA and parC confer resistance to fluoroquinolones in *Salmonella* and *Campylobacter* [112]. Ribosomal target alterations, such as methylation of 23S rRNA, contribute to macrolide resistance. Colistin resistance mediated by mcr genes (e.g., *mcr-1*) has emerged globally. These genes encode a phosphoethanolamine transferase that modifies lipid A, reducing colistin binding and efficacy. They are plasmid-borne and widespread in Enterobacterales from food sources [113].

Integrons and transposons facilitate the capture and spread of multiple resistance genes. Class 1 integrons are commonly identified in *Salmonella* and *E. coli* from animal and food sources, often linked to multidrug resistance [114]. Environmental co-selection mechanisms are gaining attention. Disinfectant use and heavy metal exposure in food production environments may co-select for AMR, enhancing efflux activity and persistence of resistance genes. Understanding these mechanisms is crucial for guiding surveillance strategies, designing targeted diagnostics, and developing effective AMR mitigation measures within One Health frameworks.

### 5.4. Prevention of Contamination

To reduce AMR contamination in foods, integrated farm-to-fork interventions are essential and must involve coordinated actions at every stage of the food production chain. On the farm level, the adoption of good agricultural practices and good veterinary practices plays a fundamental role. These practices emphasize the prudent and responsible use of antimicrobials, limiting their use to genuine clinical needs, and promoting alternatives such as vaccination, enhanced biosecurity, improved housing conditions, and better nutrition to reduce disease burden [30]. Biosecurity measures are also vital for limiting the spread of AMR. These include controlling access to farms, enforcing strict sanitation protocols for equipment and personnel, providing clean feed and water, and ensuring that the introduction of new animals or materials does not bring resistant bacteria into the farm environment [115]. Regular monitoring of antimicrobial use and resistance patterns supports early detection of emerging threats and informs better management practices.

At the slaughterhouse and food processing levels, the implementation of Hazard Analysis and Critical Control Point systems and adherence to good hygiene practices are indispensable. These systems help identify critical points where contamination could occur and set out measures to prevent or control hazards, including those related to AMR [98]. Proper cleaning and disinfection of equipment, maintenance of processing lines, and staff training in hygienic handling all contribute to minimizing cross-contamination. Finally, education of food handlers, retailers, and consumers about safe food handling, appropriate cooking practices, and the risks associated with AMR is an important supporting measure to limit the spread of resistant bacteria from foods to humans.

### 5.5. Detection Methods for AMR in Foods

Advances in diagnostic technologies now enable the rapid, sensitive, and precise detection of AMR determinants in various food matrices, supporting surveillance, outbreak investigations, and risk assessments. Among molecular tools, quantitative real-time PCR (qPCR) remains one of the most widely used methods for detecting and quantifying specific resistance genes, such as *blaCTX-M*, *mecA*, and *mcr-1*, with high specificity and sensitivity [116]. qPCR’s advantages include rapid turnaround time, cost-effectiveness, and applicability to high-throughput screening, though it is limited to detecting known target genes. For comprehensive profiling, WGS is increasingly employed to characterize the full resistome of foodborne pathogens, providing detailed insights into resistance genes, mobile genetic elements, and their potential for horizontal gene transfer. WGS is now a cornerstone in national and international AMR surveillance programs, enabling source attribution and monitoring of resistance trends [117].

Metagenomic approaches allow for culture-independent detection of AMR genes in complex microbial communities, including those present in ready-to-eat foods, raw produce, or environmental samples associated with food production. These methods provide a holistic view of both culturable and unculturable organisms and the resistome they carry, making them powerful tools in risk assessment frameworks [118]. Emerging biosensor technologies, such as electrochemical sensors and nanomaterial-based platforms, are under active development for on-site AMR detection in food products. These methods offer the promise of rapid, point-of-care screening with minimal sample preparation and could play a key role in future food safety monitoring systems [119]. Together, these complementary approaches greatly enhance our ability to detect, monitor, and respond to AMR contamination along the food chain.

### 5.6. Legislation and Policy

Policy frameworks at national and international levels are essential for addressing AMR in food systems, as they provide the regulatory backbone for surveillance, antimicrobial use, and risk mitigation. At the global level, integrated approaches based on the One Health concept are increasingly recognized as critical to tackling AMR across human, animal, and environmental interfaces [29]. This includes establishing clear guidelines and standards for antimicrobial use in food-producing animals and promoting harmonized monitoring and reporting systems. In the EU, efforts to address AMR include the promotion of integrated surveillance and risk management strategies that span human health, veterinary, and food production sectors. This approach aims to reduce antimicrobial use in agriculture while strengthening food safety and security [120]. National action plans in countries such as Thailand and the United Kingdom explicitly link AMR mitigation strategies to food safety objectives, combining regulatory controls with incentives for best practices, surveillance, and public awareness initiatives [15].

The development and enforcement of such policies require sustained commitment, inter-sectoral collaboration, and adequate resources to ensure meaningful reductions in AMR risks along the food chain. A synthesis of recent studies (2018–2024) on the prevalence of antimicrobial-resistant pathogens in various food products across different regions is presented in Table 2. These studies illustrate the global distribution of MDR strains in meat, poultry, ready-to-eat foods, aquaculture products, and animal sources, highlighting the widespread nature of AMR and its direct relevance to food safety and security. The table summarizes key findings regarding resistance patterns, food types, and geographical coverage, supporting the critical need for integrated surveillance and control measures.

### 5.7. Implications for Food Security

AMR in foodborne pathogens presents a serious and growing threat to global food security. Contamination of food products with MDR bacteria can lead to supply chain disruptions through product recalls, trade bans, and loss of consumer confidence, directly reducing the availability of safe food [99,103]. The economic consequences of AMR-related food safety incidents, including increased production costs and market losses, disproportionately affect vulnerable populations and limit equitable access to nutritious foods, particularly in LMICs [15,100].

Furthermore, AMR reduces the effectiveness of veterinary treatments essential for animal health and productivity, contributing to lower yields and threatening the stability of food systems. Food utilization is also impacted, as AMR-related foodborne infections increase illness burden, impair nutrient absorption, and elevate healthcare costs [102]. The persistence of AMR in the food chain exacerbates vulnerabilities during crises (e.g., pandemics, natural disasters), further undermining the stability pillar of food security. Addressing AMR is therefore essential not only for food safety but also as a cornerstone for ensuring resilient, equitable, and sustainable food systems. This calls for coordinated One Health actions that integrate AMR mitigation into food security, agriculture, and public health policies at local, national, and global levels [29].

Foodborne AMR threatens not only infectious disease control but also nutritional security, particularly in vulnerable populations. Outbreaks associated with AMR pathogens, precautionary culling of livestock, and trade restrictions can reduce the availability and affordability of animal-source foods. For example, Grace [98] highlighted how livestock-associated AMR reduces productivity and increases costs in low-income countries, indirectly impacting protein access. Van Boeckel et al. [30] demonstrated that rising AMR trends in food animals correlate with increased production costs and trade restrictions, affecting protein supplies. Additionally, Schar et al. [121] emphasized that AMR-related restrictions on aquaculture products in Asia have affected fish availability in local diets, contributing to nutritional insecurity. These nutritional shocks are compounded in settings where alternative protein sources are scarce or unaffordable. Foodborne AMR thus represents a dual threat to public health and food security, underscoring the need for integrated AMR mitigation strategies that also safeguard equitable nutrition.

## 6. Strategic Priorities and Challenges in One Health AMR Mitigation

### 6.1. Operationalizing One Health: Bridging Policy and Practice

The One Health approach is widely recognized as a crucial strategy to address AMR, yet translating it into operational frameworks remains a challenge [122]. In LMICs, fragmented governance, limited resources, and weak surveillance infrastructure hinder multisectoral integration. Cella et al. [122] highlight that national action plans often struggle with implementation due to a lack of political prioritization and institutional capacity. Even when action plans are in place, many countries lack the mechanisms to translate strategic goals into enforceable legislation, coordinated inter-agency action, or sustainable funding models. Moreover, institutional silos between ministries of health, agriculture, and environment often lead to parallel, uncoordinated efforts that undermine the spirit of One Health. Strengthening inter-ministerial coordination through dedicated One Health platforms and policy coherence mechanisms is essential. Capacity-building efforts must also prioritize the training of personnel in multisectoral thinking, data sharing, and integrated risk assessment. Successful examples, such as Denmark’s integrated surveillance model [123], demonstrate that cross-sectoral collaboration, stakeholder engagement, and consistent investment are key enablers. Tailoring such models to fit local governance contexts can help improve implementation and ownership at the national level.

### 6.2. Innovation and Equity: Addressing the Access Gap

While advances in diagnostics, bioinformatics, and therapeutics have opened new avenues for AMR mitigation, these innovations are largely concentrated in high-income countries. In LMICs, for example, limited access to rapid molecular diagnostics significantly delays pathogen identification and susceptibility testing, leading to empirical broad-spectrum antibiotic use and poorer clinical outcomes. A case study from Kenya revealed that only 15% of tertiary hospitals had functioning microbiology laboratories capable of culture and sensitivity testing, forcing clinicians to prescribe antibiotics based on syndromic diagnosis rather than confirmed data [124]. Similarly, a WHO-supported assessment in Southeast Asia found that genomic surveillance capacity was either absent or underutilized in over 70% of surveyed laboratories, resulting in critical delays in resistance tracking and outbreak containment [125].

Organizations such as the GARDP and the WHO Global Access Initiative for New Antibiotics (SECURE) initiative advocate for equitable access to life-saving antibiotics and diagnostics. A recent study published in The Lancet Infectious Diseases revealed that fewer than 7% of people with severe drug-resistant infections in LMICs receive the antibiotics they need [81]. This lack of access not only causes significant suffering and high mortality but may also contribute to the growing threat of AMR. The study, which examined data from eight countries, found a severe shortfall in appropriate antibiotic treatments, with access ranging from just 0.2% in Kenya to 14.9% in Mexico and Egypt [126]. Experts argue that global strategies must balance antibiotic innovation with broader access, especially in poorer nations. Drawing parallels to HIV treatment efforts, researchers have called for treatment targets and global action to improve antibiotic accessibility. To address these disparities, it is essential to implement policies that promote both the development of new antimicrobial agents and the equitable distribution of existing ones. This includes investing in healthcare infrastructure, enhancing laboratory capacities, and ensuring that LMICs have the necessary resources to combat AMR effectively.

### 6.3. Enhancing Surveillance: Integrating Environmental Data

Integrated surveillance is essential to capture the full spectrum of AMR across human, animal, and environmental domains. However, most national and international surveillance systems still primarily focus on human and veterinary sectors, neglecting the critical role of environmental reservoirs. Environmental compartments—such as surface waters, wastewater, soils, and sediments—serve as hotspots for antibiotic residues and ARGs originating from healthcare facilities, pharmaceutical manufacturing, and agricultural runoff [11]. Recent studies have highlighted the global spread of ARGs through aquatic environments. For instance, Hendriksen et al. [32] demonstrated the use of metagenomics in wastewater-based surveillance across 60 countries, revealing diverse resistance genes including those for last-resort antibiotics such as colistin. This approach offers a scalable, cost-effective way to monitor resistance trends and complements clinical surveillance data. Xiong et al. [127] further emphasized that fecal contamination and poor sanitation infrastructure significantly exacerbate environmental AMR dissemination, especially in LMICs.

The Joint Programming Initiative on AMR (JPIAMR) and WHO have both called for harmonized environmental surveillance protocols to integrate AMR tracking across sectors [95,128]. While progress has been made in some regions—such as the inclusion of waterborne AMR in the European AMR surveillance network (EARS-Net)—broader implementation remains limited. In low-resource settings, environmental surveillance faces challenges including limited laboratory capacity, lack of standardized indicators, and fragmented data sharing mechanisms [129]. To close this gap, environmental AMR surveillance should be incorporated into National Action Plans (NAPs) and supported through international funding platforms such as the Fleming Fund. Countries like Sweden and the Netherlands have established successful wastewater AMR monitoring programs that could serve as models for global adaptation [79]. Ultimately, a One Health-aligned surveillance system must integrate human, animal, and environmental data to enable the timely detection of resistance hotspots, guide policy interventions, and promote sustainable antimicrobial use practices.

### 6.4. From Awareness to Behavior Change

Raising public and professional awareness about AMR is a foundational intervention, but awareness alone does not ensure behavioral change. Chukwu et al. [130] conducted a systematic review showing that while awareness campaigns often increase knowledge, they have limited impact on changing prescribing behaviors without structural support. Similarly, Haenssgen et al. [131] found that in Southeast Asia, increased antibiotic knowledge did not translate into reduced demand for antibiotics, highlighting the importance of context-specific behavioral interventions. Horwood et al. [132] emphasized the role of peer influence, benchmarking, and real-time prescribing feedback in improving clinician adherence to antimicrobial guidelines. In a randomized trial, In a randomized trial, Meeker et al. [133] demonstrated that integrating behavioral nudges into electronic health record systems led to measurable improvements in prescribing quality. Verma et al. [134] documented the positive impact of incorporating AMR education into undergraduate medical and veterinary curricula across India, noting a significant increase in knowledge retention and reported stewardship practices. Finally, Sirota et al. [135] concluded that AMR education programs grounded in social and behavioral science were more likely to result in sustained behavioral change compared to traditional lecture-based approaches.

### 6.5. Strengthening Global Policy Frameworks

Global agencies such as the WHO, FAO, and the WOAH have developed strategic frameworks to guide AMR mitigation efforts [67,91]. These frameworks promote multisectoral collaboration, capacity building, and integrated surveillance systems based on the One Health concept. However, national uptake of Codex guidelines and related global action plans is uneven [67,136]. The ACT project by FAO helps countries adapt global AMR standards to local contexts, offering technical assistance, training, and policy guidance. A notable example is Thailand’s National Strategic Plan on AMR (2017–2021), which incorporated WHO guidance and established coordination among ministries of public health, agriculture, and environment. The plan resulted in measurable reductions in antibiotic use in humans and animals, supported by robust surveillance and community engagement.

Similarly, Sweden has demonstrated the effectiveness of sustained investment and cross-sectoral coordination in curbing AMR through the Swedish Strategic Programme Against Antibiotic Resistance (STRAMA) initiative. STRAMA has led to a significant decline in antibiotic prescriptions and improved public awareness through educational campaigns and strict prescribing regulations. Multilateral commitments, such as those made by the G7 and the United Nations, have elevated AMR on the global health agenda [137,138], but sustained political will and investment are required to operationalize these commitments at the country level. Integrating global recommendations into national action plans, ensuring multisectoral stakeholder buy-in, and establishing monitoring frameworks are critical steps for long-term policy effectiveness. To facilitate a clearer understanding of the conclusions drawn in this review, Table 3 presents a structured summary of the main thematic areas explored, the corresponding key findings, and strategic recommendations. This table underscores the multifaceted nature of foodborne AMR and provides a concise roadmap for researchers, policymakers, and public health professionals working to address this global threat through the One Health approach.

## 7. Conclusions

The convergence of microbial food safety and AMR presents a formidable and evolving threat to global health, food security, and sustainable development. This review has elucidated the multifaceted nature of foodborne AMR, highlighting critical points of emergence, transmission, and amplification across human, animal, and environmental domains. The widespread use and misuse of antibiotics in agriculture, aquaculture, and human medicine have facilitated the persistence and dissemination of MDR pathogens such as *Salmonella* spp., *E. coli*, *L. monocytogenes*, and *Campylobacter* spp. throughout the food chain, especially in RTE products. Tackling this crisis requires a robust One Health approach that integrates antimicrobial stewardship, surveillance, regulation, innovation, and public education. Advances in molecular diagnostics, whole-genome sequencing, and resistome profiling have been improving our ability to detect, monitor, and predict resistance patterns, while alternative interventions such as phage therapy, bacteriocins, and probiotics offer promising tools for reducing antibiotic dependence. However, innovation must be matched by equitable access—particularly in LMICs where laboratory capacity, surveillance infrastructure, and treatment availability remain limited. Sustained political commitment, global policy harmonization, and integrated action plans are essential for operationalizing the One Health framework. Embedding environmental monitoring, regulating antibiotic use in food systems, and fostering behavioral change through education will be key to slowing the tide of AMR. As the food system continues to globalize, a coordinated international response is critical to safeguard both human health and food safety in the face of rising resistance. These findings underscore the need for integrated, farm-to-fork interventions, robust surveillance systems, and policy frameworks that address AMR risks in foods. In conclusion, tackling AMR in food systems is integral to safeguarding food security and requires harmonized One Health strategies spanning agriculture, food safety, veterinary care, and public health.

## Figures and Tables

**Table 1:** Key national and international agencies and surveillance programs involved in foodborne AMR mitigation, summarizing their main roles and interactions within One Health frameworks.

Agency/Program	Key Role(s)	Coordination and Interaction
WHO	Oversees the GLASS, which standardizes AMR and antimicrobial consumption data—including for food and environment—across participating countries.	Works with FAO, WOAH, and United Nations Environment Programme (UNEP) under the Quadripartite/Tripartite One Health alliance.
FAO	Implements the Progressive Management Pathway for AMR (PMP-AMR) to support agriculture in managing AMR and food safety.	Engages in One Health collaboration with WHO and WOAH and supports national AMR Action Plans.
WOAH	Develops veterinary antimicrobial use standards, monitors AMR in animals, and promotes stewardship.	Partners with WHO, FAO, and member countries through One Health initiatives.
GLASS	Collects harmonized data on AMR and antimicrobial consumption from human, animal, and environmental sectors.	Integrates regional networks (e.g., European Antimicrobial Resistance Surveillance Network (EARS-Net), Central Asian and Eastern European Surveillance of Antimicrobial Resistance) and feeds data to WHO.
EFSA	Monitors AMR in food- and animal-origin samples within the EU.	Coordinates with European Centre for Disease Prevention and Control (ECDC) to support EU risk assessments and policy.
ECDC	Tracks AMR in human pathogens through EARS-Net and contributes to EU-level surveillance.	Collaborates with EFSA and national agencies under One Health.
NARMS	Monitors AMR in human, food, and animal bacterial samples via a One Health approach.	Led jointly by CDC, FDA, and USDA; supports data-driven interventions.
CDC Global AMR Lab and Response Network	Strengthens AMR detection capacity and rapid response through global lab networks.	Partners with international agencies; supports ~50 countries.
Global Leaders Group on AMR	Provides high-level advocacy and policy guidance to spur AMR action with a One Health lens.	Supported by a Quadripartite secretariat (WHO, FAO, WOAH, UNEP).

**Table 2:** Recent studies (2018–2024) on the prevalence of antimicrobial-resistant pathogens in food products across different regions.

Study (Author, Year)	Country/Region	Food Type	Key Findings on AMR
Tang et al. [99]	China	Retail pork and chicken meat	13% *E. coli* carried *mcr-1*; most co-produced ESBLs, indicating co-resistance to critical antibiotics
Nhung et al. [100]	Vietnam	Retail meat	>50% *Salmonella* MDR; high resistance to fluoroquinolones and ESBLs
Adel et al. [103]	Egypt	Retail meats and slaughterhouse isolates	82.4% *S. enterica* MDR; 41.2% ESBL producers; 67.6% carried plasmid-mediated quinolone resistance genes (*qnrA*, *qnrB*, *qnrS*)
Kayode & Okoh [102]	South Africa	RTE foods	83.5% *L. monocytogenes* MDR; 53% resistant to ceftriaxone; 61.9% to trimethoprim; 62.9% to oxytetracycline
Bort et al. [104]	Spain	Broiler production chain	97.6% *Campylobacter* resistant to ≥1 antibiotic; high resistance to fluoroquinolones and macrolides
Shafiq et al. [44]	Pakistan	Food-producing animals	ESBL-producing, colistin-resistant *E. coli* isolated from healthy animals
Zhang et al. [42]	China	Retail aquatic products	High prevalence of MDR *E. coli*; first report of *mcr-1*-positive ESBL-producing *E. coli* ST2705 and ST10 in fish

**Table 3:** Summary of Major Findings and Strategic Recommendations for Combating Foodborne AMR.

Thematic Area	Key Finding	Reference	Strategic Recommendation	Reference
Microbial Food Safety and AMR Threats	High prevalence of MDR Salmonella, *E. coli*, *L. monocytogenes*, and *Campylobacter* in foods, especially ready-to-eat products across the EU	[41]	Implement farm-to-fork microbiological monitoring programs targeting RTE and minimally processed foods	[139]
Antibiotic Use in Food Production	Non-therapeutic use of antimicrobials (e.g., growth promotion) is a major AMR driver in livestock	[30]	Ban prophylactic antimicrobial use, enforce veterinary oversight, and promote stewardship in animal sectors	[139]
Environmental Reservoirs	Sewage and agricultural runoff harbor diverse AMR genes, reflecting environmental contamination	[32]	Integrate environmental samples (wastewater, manure, soil) into AMR surveillance; enhance pollution control	[139]
One Health Implementation	Fragmented governance hinders multisectoral coordination in AMR control	[122]	Establish inter-ministerial One Health coordination platforms and capacity building in risk analysis	[122]
Surveillance Gaps	Underrepresentation of animal and environmental data in national AMR systems	[41]	Broaden AMR surveillance to include metagenomic and wastewater approaches; support LMIC implementation	[32]
Innovation and Access Inequalities	Aquaculture AMU is rising (~93,000 t in 2017, projected +11.5% by 2030); LMIC diagnostics remain limited	[30,140]	Invest in affordable diagnostics in LMICs; support regional labs and rapid molecular tools	[140]
Behavior Change and Public Awareness	AMR awareness campaigns alone often fail to change prescribing behavior	[141]	Integrate behavioral nudges, peer comparison, and electronic prescribing feedback in stewardship	[133]
Policy and Governance Challenges	Global AMR strategies have variable national implementation; funding remains inadequate	[142]	Support local adaptation; embed AMR plans into national health and development agendas; secure sustainable financing	[141]

## Data Availability

No new data were created or analyzed in this study. Data sharing is not applicable to this article.
